# Monthly Summary

**Published:** 1871-12

**Authors:** 


### MONTHLY SUMMARY.

---

*Dentistry in the United States.*—The dental profession in the United States, it is understood comprises 13,000 members, who earn an aggregate income of $24,000,000. The annual expenditure for materials amounts to $2,000,000, and for office rents to $3,000,000, leaving $19,000,000 as the net income of the profession. There are 9 dental colleges in the United States, from which 1,807 persons have graduated. The profession supports several dental periodicals, with an aggregate circulation of 12,900 copies. There are no dental colleges in the countries of Europe. Dentistry previous to the present century did not occupy the position of a profession, but its practitioners, it is said, were to a great extent quacks. In consequence of the greater skill of dentists of the present day, the tendency of the practice, it is stated, is to save the natural teeth and to avoid the necessity of inserting artificial teeth.—*Exchange.*

---

*A Useful Varnish.*—Three parts of blood deprived of its fibrine, four of lime, and a little alum, form a composition which, when applied to cardboard, renders it as hard as wood, and thoroughly impervious to water. It is a Chinese preparation, many of the houses in Pekin being coated with it.—*Med. and Surgical Reporter.*

*Ancient Dentistry.*—Dr. Reid, of Terre Haute, read a paper upon ancient dentistry. Among the ancients great success was obtained in this art. Casselius was a dentist in the reign of the Roman triumvirs, and gold was used for the filling. But nearly 500 B. C., gold was thus used, and gold wire was employed to hold artificial teeth in position, and it does not seem then to have been a new art. A fragment of the tenth of the Roman tables, 450 B. C., has reference to the burial of any gold with the dead except that bound around the teeth. Heroditus declares that the Egyptians had a knowledge of the diseases of teeth and their treatment 2,000 B. C. In Martial, Casselius is mentioned as either filling or extracting teeth ; but he specified that he would not polish false teeth with tooth powder. Lucian mentions an old maid that had but four teeth, and they were fastened in with gold. These facts cover a period of 600 years. —*Med. and Surg. Rep.*

---

*The Protective Power of Vaccination.*—During these times of skepticism as to the protective virtue of vaccination, it may be of some interest to learn a little fact which has lately come to our knowledge, on the accuracy of which perfect reliance can be placed. A military surgeon having recently had occasion to examine a large number of English recruits, found that 60 per cent. of lads unprotected by vaccination had been the subjects of small-pox, as against 1.90 per cent. of protected recruits who bore no traces of small-pox. Remembering that these are men who have escaped any permanent damage from the disease, what must be the percentage of attacks in unprotected cases generally? —*Lancet.*

---

*Anæsthetics—Their Relative Safety.*—From a careful examination of the statistics of 209,893 cases, Prof. E. Andrews gives, in the *Chicago Medical Examiner*, the following estimate of the relative danger from different anæsthetics :

| | | | | | |
|---|---|---|---|---|---|
| Sul. Ether.................... | 1 | death | to | 23,204 | administrations. |
| Chloroform...................... | 1 | " | " | 2,723 | " |
| Mixed Chlo. and Ether.. | 1 | " | " | 5,588 | " |
| Bichloride of Methylene | 1 | " | " | 7,000 | " |
| Nitrous Oxide................ | No deaths in | | | 75,000 | " |

—*Dental Cosmos.*

